# How mimicry influences the neural correlates of reward: An fMRI study

**DOI:** 10.1016/j.neuropsychologia.2017.08.018

**Published:** 2018-07-31

**Authors:** Chun-Ting Hsu, Thomas Sims, Bhismadev Chakrabarti

**Affiliations:** Centre for Integrative Neuroscience and Neurodynamics, School of Psychology and Clinical Language Sciences, University of Reading, Whiteknights, Reading RG6 6AL, UK

**Keywords:** Facial mimicry, Social reward, Empathy, Interpersonal Reactivity Index

## Abstract

Mimicry has been suggested to function as a “social glue”, a key mechanism that helps to build social rapport. It leads to increased feeling of closeness toward the mimicker as well as greater liking, suggesting close bidirectional links with reward. In recent work using eye-gaze tracking, we have demonstrated that the reward value of being mimicked, measured using a preferential looking paradigm, is directly proportional to trait empathy (Neufeld and Chakrabarti, 2016). In the current manuscript, we investigated the reward value of the act of mimicking, using a simple task manipulation that involved allowing or inhibiting spontaneous facial mimicry in response to dynamic expressions of positive emotion. We found greater reward-related neural activity in response to the condition where mimicry was allowed compared to that where mimicry was inhibited. The magnitude of this link from mimicry to reward response was positively correlated to trait empathy.

## Highlights

• Mimicry is a core feature of human social interaction, and builds social rapport.
• Mimicry arguably helps build social rapport through its links with the reward system.
• Spontaneous facial mimicry was restricted (or not) in this fMRI experiment.
• Reward-related neural response was greater when mimicry was unrestricted.
• Reward-related neural response to mimicry was proportional to trait empathy.

## 1 Introduction

Mimicry is a facilitator of social bonds in humans. Spontaneous mimicry of facial expressions of emotion is seen in humans from an early stage in development, and contributes to the affective response to another person's emotion state, i.e. affective empathy (Meltzoff, 2007, Meltzoff and Decety, 2003, Meltzoff and Moore, 2002). Social psychological studies have suggested a bidirectional link between mimicry and liking. Human adults like those who mimic them, and mimic others more who they like (Kühn et al., 2010, Likowski et al., 2008, McIntosh, 2006, Stel and Vonk, 2010; Lakin et al., 2003). Liking and affiliation goals can be regarded as complex social processes that effectively alter the reward value of social stimuli. Consistently, experimentally manipulating the reward value associated with a face influences the extent of its spontaneous mimicry (Sims et al., 2012). At a neural level, functional connectivity between brain areas involved in reward processing (ventral striatum, VS) and facial mimicry (inferior frontal gyrus, IFG) was found to be higher when observing faces conditioned with high vs. low reward (Sims et al., 2014). Using an identical paradigm in an EEG experiment, greater mu-suppression (related to mimicry-relevant sensorimotor coupling/ mirror system activity) was noted in response to faces associated with high vs. low reward (Trilla-Gros et al., 2015).

The link from reward to mimicry is relevant to understand social communication in individuals who score low on measures of trait empathy, such as those with Autism Spectrum Disorder (ASD). Individuals with ASD display reduced spontaneous mimicry for the emotional facial expressions of others (Beall et al., 2008, McIntosh et al., 2006, Oberman et al., 2009). One hypothesis suggests that such reduced spontaneous facial mimicry is driven, in part, by the low reward value ascribed to faces and other social stimuli in individuals with ASD (Dawson et al., 2002; Chevallier et al., 2012). Consistent with this hypothesis, the link from reward to mimicry has been shown to be weak in individuals with high autism-related traits (Sims et al., 2014, Sims et al., 2012). Crucially however, the link between reward and mimicry is bidirectional. It is important to study these links in both directions, since mimicry is a key component of human behaviour from early development, and such bidirectional links with reward provides a potential mechanism through which mimicry facilitates social bonds.

If mimicry is rewarding by nature, two possibilities arise. First, the act of being mimicked is rewarding. Behavioural studies support this possibility by demonstrating that individuals find being mimicked to be more rewarding (Neufeld and Chakrabarti, 2016, van Baaren et al., 2004). Greater self-reported liking and reward-response (indexed by preferential gaze duration) was associated with faces that show greater mimicry vs. those that show lower mimicry (Neufeld and Chakrabarti, 2016). Importantly, the strength of this link from mimicry to reward was greater in individuals with high trait empathy. Second, that the act of mimicking *itself* is rewarding to the mimicker, as suggested from observations in non-human primates (de Waal and Bonnie, 2009). There is little or no empirical investigation of this second possibility. In order to fill this gap in the literature, we investigated the effect of inhibiting spontaneous facial mimicry on the extent of reward processing.

A commonly used technique to restrict spontaneous mimicry of happy facial expressions is to interfere with a participant's capacity to smile, by having him/her hold a pen between their lips. This action contracts the orbicularis oris muscle complex that surrounds the mouth and is incompatible with the contraction of the zygomaticus major muscle group in the cheek that is needed for smiling (Strack et al., 1988). Niedenthal (2007) and colleagues showed that happy faces were rated as less positive when participants’ ability to spontaneously mimic was restricted using the procedure described above. We sought to use this manipulation as a potential method to restrict spontaneous mimicry of happy expressions. For the task to be suitable for use in the MRI scanner, we modified the task to instruct the participants to hold their tongue between their lips for half of the trials. This condition is referred to as the “Tongue” condition. In the remaining trials participants were merely instructed to observe the stimuli that were presented. This condition is referred to as the “NoTongue” condition. We performed a pilot study using facial EMG in order to validate the effectiveness of the method to restrict facial mimicry (described in Section 2). Notably, this ‘Tongue’ vs. ‘NoTongue’ manipulation does not have any impact on the mimicry of angry faces, which needs the free movement of the corrugator supercilii muscle.

The aim of the main study was to measure the response of two key brain regions involved in processing rewards - (ventral striatum [VS] and orbitofrontal cortex [OFC]) - as participants observed happy and angry facial expressions under two conditions, that either allowed or restricted spontaneous facial mimicry of happy faces.

The VS receives cortical input from the OFC and anterior cingulate cortex, as well as mesolimbic dopaminergic afferents. It projects back to the ventral tegmental area and substantia nigra, which, in turn, have projections to the prefrontal cortex, via the medial dorsal nucleus of the thalamus (Haber and Knutson, 2010). This circuit is an integral part of the cortico-basal ganglia system and plays a central role in reward processing in humans and other mammals. The OFC is another key node of this circuit, and is believed to encode the subjective value of stimuli, as suggested by multiple studies in humans and nonhuman primates (Rolls, 2000, Wallis, 2011). OFC neurons in primates have been shown to be involved in social context-dependent coding of reward value (Azzi et al., 2012). Activity in VS has been suggested to be related to the anticipation of both primary and secondary rewards, while OFC potentially serves to encode a variety of stimuli into a common currency in terms of their reward values (Haber and Knutson, 2010, Liu et al., 2011; O'Doherty, 2004; O'Doherty et al., 2002; Schultz et al., 2000).

We hypothesised that spontaneous mimicry of happy facial expressions would evoke greater activity in the VS and OFC compared to the condition where spontaneous mimicry is restricted. This hypothesis relies on the assumption of a feedforward signal from the brain areas involved in the act of mimicry to those involved in the reward response. This assumption is supported by a previous fMRI study, where activity in the parietofrontal network involved in mimicry in response to observing another human making an action toward an object was found to modulate the reward-related neural response to the object, as well as the self-reported desirability of the object (Lebreton et al., 2012). Increased striatal activity has also been shown whilst participants intentionally mimic, as opposed to merely observe, emotional facial expressions (Carr et al., 2003). Activity in the VS and the OFC during mimicry of hand signals has been shown to be modulated by “similarity biases” such as gender (Losin et al., 2012). However, the impact that spontaneous facial mimicry has on brain regions involved in reward processing has not been directly tested.

In order to test the effectiveness of the proposed mimicry manipulation, it is necessary to measure the IFG response, while spontaneous mimicry was allowed or restricted. IFG activity has been repeatedly associated with mimicry, as demonstrated in a meta-analysis (Caspers et al., 2010). The control condition involved participants’ viewing angry facial expressions. As the spontaneous mimicry of angry faces requires sets of muscles that should not be inhibited during the Tongue condition (e.g. the corrugator supercilii) we would not expect to see any difference in IFG activity between the NoTongue and Tongue conditions in response to angry faces. We predicted that

i) a significant Tongue × Emotion interaction will be observed in the VS and the OFC response. Specifically, greater BOLD activity was predicted in the VS and OFC in response to NoTongue (High Spontaneous Mimicry) Happy vs. Tongue (Low Spontaneous Mimicry) Happy faces, but not in response to NoTongue Angry vs. Tongue Angry faces;
ii) a significant Tongue × Emotion interaction will be observed in IFG. Specifically, greater BOLD activity was predicted in the IFG in response to NoTongue Happy vs. Tongue Happy faces, but no difference in response to NoTongue Angry vs. Tongue Angry faces.

Individual differences in the strength of the link from mimicry to reward are of particular interest, in light of a previous study which demonstrated that individuals high in trait empathy showed a greater liking and preferential looking for faces who mimicked them more (Neufeld and Chakrabarti, 2016). Accordingly, a widely used and well-characterised trait measure of empathy, the Interpersonal Reactivity Index was used in the current study (IRI: Davis, 1980, Davis, 1983). Of specific interest was the correlation between individual differences in empathy and reward response to [free vs restricted-mimicry] happy faces in reward-related brain regions (VS and OFC). Based on previous human studies, we predicted that participants’ IRI score would correlate positively with the Tongue x Emotion interaction term of the BOLD response in the reward-related regions.

## 2 Material and methods

Ethical approval for the pilot validation study and the main fMRI study was obtained from the University Research Ethics Committee of the University of Reading and all participants provided informed consent.

### 2.1 Pilot study: Validation of the manipulation to restrict facial mimicry

Six participants (4 female) with normal or corrected-to-normal vision were recruited from the University of Reading campus. Participants viewed movie clips of actors making either happy or angry facial expressions in two conditions (“Tongue” and “NoTongue”). The visual presentation and the EMG measurement were the same as in Sims et al. (2012). However, sensors were placed only over the zygomaticus major muscle. As in Sims et al. (2012), EMG data was rectified, screened for movement artefacts, and logarithmically transformed. The baseline for each trial was defined as the mean magnitude in activity for the period 500 ms prior to stimulus onset. The mean EMG magnitude for the period 2000–4000 of stimulus presentation was then calculated, and then divided by the pre-stimulus baseline (De Wied et al., 2009). A 2 (emotion: happy, angry) × 2 (mimicry conditions: Tongue, No Tongue) repeated measures ANOVA was performed. Of interest were specific pairwise comparisons, namely [NoTongue Happy vs. Tongue Happy], [NoTongue Angry vs. Tongue Angry], [Tongue Happy vs. Tongue Angry] and [NoTongue Happy vs. NoTongue Angry] to detect if the Tongue/NoTongue manipulation significantly and specifically restricts spontaneous mimicry of happy faces.

### 2.2 Main fMRI study

#### 2.2.1 Participants

Twenty-nine neurotypical participants (17 females) aged between 20 and 36 years (mean age ± SD = 22.96 ± 4.17) were recruited from the University of Reading campus. Participants received an anatomical image of their brain in exchange for their participation. All participants had normal or corrected to normal vision.

#### 2.2.2 Stimulus materials

Stimuli consisted of dynamic clips of ten actors (5 male, 5 female) each with two different facial expressions; happy and angry. Each clip lasted 1800 ms. All stimuli were selected from the STOIC set which have been shown to have high inter-rater reliability (Roy et al., 2007, available at http://www.mapageweb.umontreal.ca/gosselif/cv.html). The images were displayed using E-Prime 2.0 (Psychology Software Tools, PA, USA) and were presented using Nordic NeuroLab's VisualSystem goggles (Nordic Neurolab Inc, WI, USA).

#### 2.2.3 Procedure

On arriving at the lab, participants were introduced to the task outside of the scanner. The experimenter demonstrated holding his tongue between his lips in the manner that was required of the participant. To minimise movement during the trials, participants were asked to get their tongue into position as quickly as possible upon seeing the text instruction on screen. Participants completed four practice blocks outside the scanner, one for each condition in the task. The practice blocks consisted of clips of actors taken from a separate stimulus set to that used in the recorded trials (Mindreading dataset; Baron-Cohen et al., 2004). When the experimenter was satisfied that the participant had mastered the task the participants were taken into the MRI scanner suit and were positioned inside the bore of the scanner. After completion of the task participants were debriefed and dismissed.

#### 2.2.4 Task

**Fig. 1:**
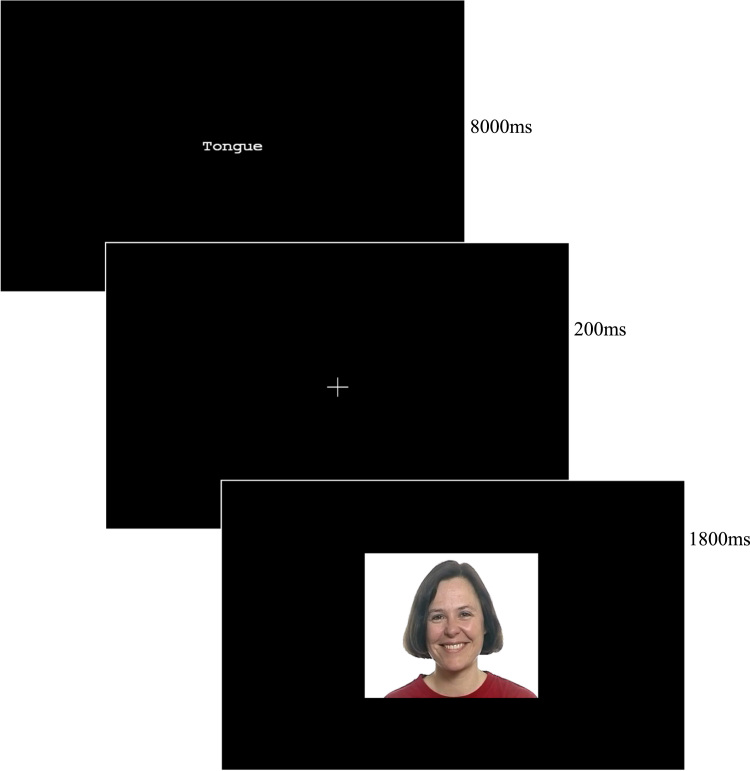
The experimental paradigm with a representative stimulus. Participants were presented with experimental blocks each consisting of eight 1800 ms video clips of faces displaying either happy or angry emotional expression. Prior to each block a single word instruction appeared on the screen; “Tongue” or “No Tongue”. In the Tongue condition participants were required to hold their tongue between their lips as they watched the faces. During the NoTongue condition participants were instructed to simply watch the faces as they appeared on screen.

The task participants had to do in the scanner had a 2 × 2 block design; the four conditions were NoTongue-Happy, Tongue-Happy, NoTongue-Angry, and Tongue-Angry. There were eight blocks for each of the four conditions. In each block, participants were presented with eight dynamic clips of faces making emotional facial expressions. There was a 200 ms blank screen immediately prior to the presentation of each clip within the block. A block lasted 16 s. Prior to each block an instruction appeared on the screen for 8000 ms. The instruction read either “NoTongue” or “Tongue”. During the Tongue condition participants were required to hold their tongue between their lips in the manner found to reduce EMG activity in the zygomaticus major in the pilot study. In the NoTongue condition participants were simply instructed to watch the clips as they were presented (to allow for spontaneous mimicry, Fig. 1).

All blocks were presented in a single run. The order of the blocks was randomised using www.randomizer.org. The order was reversed for half of the participants. The task lasted for 640 s, and a total of 330 TRs were collected.

#### 2.2.5 Regions of interest

**Fig. 2:**
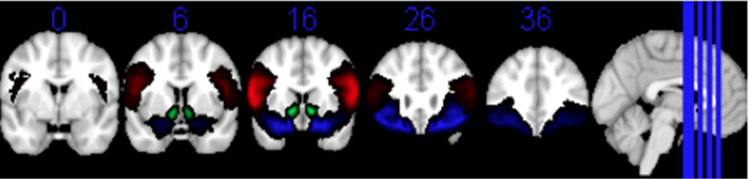
Regions of Interest. Regions of interest within the IFG (Red), VS (Green), and OFC (Blue) were defined using Harvard Oxford structural human brain atlas (Desikan et al., 2006). Voxels with a higher probability of belonging to the structure of interest are represented by lighter shades. Activation in these voxels was weighted more heavily during the ROI analysis compared with voxels with lower probability represented by darker shades.

Our regions of interest included bilateral IFG, VS and OFC (6 ROIs). All three anatomical regions have been shown to be active during mimicry tasks (Carr et al., 2003, Losin et al., 2012). Furthermore, past human and animal studies suggested that the VS and OFC are vital parts of the reward processing circuit (Haber and Knutson, 2010, Liu et al., 2011; O'Doherty, 2004; Schultz et al., 1992; Schultz et al., 2000). Independent ROIs defined by the Harvard-Oxford structural human brain atlas, as available within FSL, were used for analysis (Desikan et al., 2006; Fig. 2).

#### 2.2.6 fMRI scanning and preprocessing

Participants were scanned in a 3 T Siemens TIM Trio MRI scanner with 12 channel head coil (32 inter-leaved, 2.5 mm thick axial slices [repetition time (TR) = 2000 ms; echo time (TE) 30 ms]). Regarding the fMRI data preprocessing, the removal of movement artefacts was carried out using FEAT (FMRI Expert Analysis Tool) Version 5.98, part of FSL (FMRIB's Software Library, www.fmrib.ox.ac.uk/fsl). An independent component analysis (ICA) was performed in order to minimise the effects of any condition specific movement artefacts resulting from the Tongue task. For each participant MELODIC 3.0 was used to decompose the activation time series across the whole run into independent components. The activation maps for all components with an eigenvalue placing them before the point of inflection on a representative scree plot were visually inspected. Components strongly suspected of being movement artefacts were removed from the subsequent analysis. Examples would include components showing a “halo” of activation encircling the brain or activation in the ventricles.

The data was then further preprocessed and analyzed using the software package SPM8 (www.fil.ion.ucl.ac.uk/spm). Preprocessing consisted of slice-timing correction, realignment, and sequential coregistration. Structural images were segmented into grey matter, white matter, cerebrospinal fluid, bone, soft tissue, and air/background (Ashburner and Friston, 2005). A group anatomical template was created with DARTEL (Diffeomorphic Anatomical Registration using Exponentiated Lie algebra; Ashburner, 2007) toolbox from the segmented grey and white matter images. Transformation parameters for structural images were then applied to functional images to normalize them to the brain template of the Montreal Neurological Institute (MNI) supplied with SPM. Functional images were spatially smoothed with a kernel of 5 mm full-width-at-half-maximum after normalization.

#### 2.2.7 fMRI data analysis

Statistical parametric maps were calculated with multiple regressions of the data onto a model of the hemodynamic response (Friston et al., 1995). The first level general linear model analyses contained five regressors for “NoTongue-Happy”, “Tongue-Happy”, “NoTongue-Angry”, “Tongue-Angry”, and “Tongue Instruction” conditions. Each block in the first four conditions lasted 16 s (eight 200 ms blank screen plus eight 1800 ms videos). Each block in the “Tongue Instruction” condition lasted 8 s. Regressors were convolved with the canonical hemodynamic response function. For each ROI, the mean contrast values of the Emotion × Tongue interaction contrast [(NoTongue-Happy – Tongue-Happy) – (NoTongue-Angry – Tongue-Angry)] for each participant were extracted with Marsbar (version 0.44) and used for the group level one-sample *t*-test. 23 of the 29 participants completed the IRI. For ROIs showing significant interaction effects, mean contrast values of the interaction contrast were further used for the bivariate Pearson correlation analyses (with restricted maximum likelihood estimation) with the trait measurements. Mean ± 3 SD was used as the criteria to filter outliers, and none were identified.

For the group level analysis on the whole brain, estimates of each of the interaction contrast from each participant were used to model 1) a random effect one sample *t*-test (against a test value of 0) for the interaction contrast, and 2) a random effect multiple regression of the interaction contrast with IRI as the regressor. We imposed an initial voxel-level threshold of uncorrected p < .001, and then a cluster-level threshold of family-wise error (FWE) corrected p < .05 for the entire image volume. The anatomical labels reported in the results were taken from the Talairach Daemon database (Lancaster et al., 1997, Lancaster et al., 2000) or the AAL atlas (Tzourio-Mazoyer et al., 2002) incorporated in the WFU Pickatlas Tool (Maldjian et al., 2003). The Brodmann areas (BA) were further checked with the Talairach Client using nearest grey matter search after coordinate transformation using the WFU Pickatlas Tool.

## 3 Results

### 3.1 Pilot facial EMG study (manipulation check)

Simple pairwise comparison of conditions revealed that the zygomaticus major response was greater for NoTongue Happy vs. Tongue Happy faces, *t*(5) = 2.150, *p*_*uncorrected*_ = 0.042, *d* = 0.896, and not for NoTongue Angry vs. Tongue Angry faces, *t*(5) = − 0.250, *p*_*uncorrected*_ = 0.406. As was expected, there was significantly greater zygomaticus major activity in response to NoTongue Happy vs. NoTongue Angry faces, *t*(5) = 2.158, *p*_*uncorrected*_ = 0.042, *d* = 0.627, but not in response to Tongue Happy vs. Tongue Angry faces *t*(5) = − 0.245, *p*_*uncorrected*_ = 0.408. However, the 2 × 2 repeated measures ANOVA yielded no significant main effect of task (Tongue vs. NoTongue, *F*_*(1,5)*_ = 0.203, *p*_*Greenhouse-Geisser Corrected*_ = 0.336) and interaction between Tongue × Emotion *F*_*(1,5)*_ = 1.758, *p*_*Greenhouse-Geisser Corrected*_ = 0.121), which was possibly driven by the low sample size of the pilot study (n = 6).

### 3.2 ROI analyses and trait empathy (IRI) correlation

**Fig. 3:**
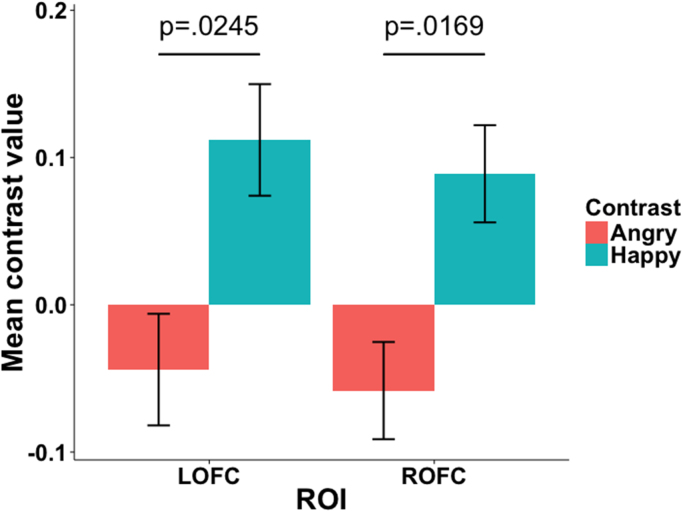
The mean values of the contrast [NoTongue - Tongue] separately for the Angry and Happy face condition in the left and right OFC. The error bars show the within-subject standard errors calculated according to Cousineau (2005). P-values of the paired *t*-test between [NoTongue-Happy – Tongue-Happy] vs. [NoTongue-Angry - Tongue-Angry] are shown.

The interaction contrast was significantly greater than zero in bilateral IFG (n = 29, LIFG mean contrast value = 0.28, *p* = 0.0004; RIFG mean contrast value = 0.24, *p* = 0.009) and bilateral OFC (LOFC mean contrast value = 0.16, *p* = 0.0245; ROFC mean contrast value = 0.15, *p* = 0.0169, Fig. 3), but not in the VS (LVS mean contrast value = 0.055, *p* = 0.2021; RVS mean contrast value = 0.02, *p* = 0.3688). As shown in Fig. 3, this interaction was due to the significant positive difference between the NoTongue Happy vs. Tongue Happy conditions, while there is no difference between the NoTongue Angry vs. Tongue Angry conditions.

**Fig. 4:**
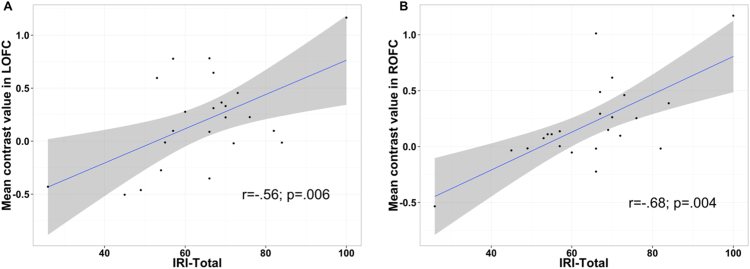
Scatter plots showing the correlations between empathy traits and the values of the interaction contrast in the left and right OFC. Panel A shows the correlation with IRI total score in the left OFC. Panel B shows the correlation in the right OFC.

Correlational analyses with trait empathy (IRI) revealed significant positive correlations between bilateral OFC and the total IRI score, (n = 23; LOFC – IRI-Total: *r* = 0.56, *p* = 0.006; ROFC – IRI-Total: *r* = 0.68, *p* = 0.0004, Fig. 4). One tailed p-values were reported, in keeping with the directional nature of the hypotheses.

### 3.3 Whole Brain Analysis and trait empathy (IRI) correlation

Significant clusters in the bilateral precentral gyrus, right superior and transverse temporal gyri, and left IFG pars triangularis were noted in response to the [(NoTongue-Happy – Tongue-Happy) – (NoTongue-Angry – Tongue-Angry)] contrast (Table 1). No significant cluster was noted for the interaction contrast in the opposite direction, i.e. [((NoTongue-Angry – Tongue-Angry)- (NoTongue-Happy – Tongue-Happy)].

**Table 1:** Task fMRI results: whole brain analysis.

**H**	**Regions**	**Cluster size**	**p (FWE)**^1^	**t statistics**	**BA**	**MNI [x, y, z]**
**Interaction [(NoTongue-Happy – Tongue-Happy) – (NoTongue-Angry – Tongue-Angry)]**						
L	Precentral gyrus	175	< 0.001	5.51	4, 6	− 57 − 15 38
R	TTG, STG, Precentral	156	< 0.001	5.3	42, 22, 4	63 − 12 15
L	IFG pars triangularis	50	0.005	4.83	46, 45	− 54 27 19
**Correlation results (Interaction contrast correlated with IRI): Whole brain analysis**						
R	TPJ: MTG, AG	59	0.001	7.68	39, 19	33 −69 19
B	Midbrain & Pons	120	< 0.001	6.57		− 6 − 30 − 22
R	dmPFC	49	0.003	6.54	9	12 36 30
L	Cerebellum	107	< 0.001	5.74		− 21 − 60 − 19
R	MCC, dmPFC	41	0.007	5.13	24, 6	12 − 3 41
R	Precentral	48	0.003	5.07	6	54 − 12 34
L	aTL: STG, MTG	28	0.043	4.77	38	− 39 15 −1 9
L	aTL: MTG	50	0.002	4.74	38, 20, 21	− 36 3 − 38

Abbreviations: H = hemisphere; B = bilateral; L = left; R = right; AG = angular gyrus; aTL = anterior temporal lobe; dmPFC = dorsomedial prefrontal cortex; IFG = inferior frontal gyrus; MCC = mid-cingulate cortex; MTG = middle temporal gyrus; STG = superior temporal gyrus; TTG = Transverse temporal gyrus; BA = Brodmann area.

* Voxel-level uncorrected p < .001, cluster-level FWE-corrected for the whole brain.

Regression analysis of the interaction contrast with the IRI score revealed significant clusters positively correlated with IRI including posterior right TPJ, mid-cingulate cortex, and precentral gyrus (full list in Table 1).

## 4 Discussion

In this study, BOLD response in two key brain regions involved in reward processing were measured, as participants observed faces making happy and angry facial expressions under two conditions. In the first condition the participants held their tongue between their lips in order to suppress spontaneous mimicry of happy facial expressions. In the second condition participants merely observed the faces, allowing for natural spontaneous mimicry. It was predicted that activation in these reward-related regions, VS and the OFC, would be greater in response to the NoTongue Happy (mimicry uninhibited) condition compared with the Tongue Happy (mimicry restricted) condition, and that this response will be positively associated with trait empathy. Our predictions were partially supported.

In the pilot study we checked whether holding the tongue restricted facial mimicry. Six individuals were tested while they viewed happy faces. We found greater zygomaticus response to happy faces when individuals were free to mimic (NoTongue condition) compared to when their spontaneous mimicry was restricted (Tongue condition). Although the Tongue × Emotion interaction was not significant due to the low statistical power resulting from the small sample size (n = 6), we concluded that the Tongue condition is a suitable scanner-friendly alternative to having participants hold a pen between their lips in the manner that has been successfully used to restrict spontaneous facial mimicry in previous literature.

We found a significant Tongue × Emotion interaction effect in the activity of bilateral IFG. This result acts as a manipulation check for the paradigm within the scanner, as it suggests that mimicry-related neural activity is reduced when spontaneous facial mimicry for the happy faces is restricted. This region also survived cluster-level correction for the interaction contrast in the whole brain analysis. Activity in the IFG has been reliably associated with action observation as well as imitation, and accordingly this region has been suggested to be part of the mirror neuron system (Caspers et al., 2010). The whole-brain analysis revealed significant clusters in the IFG as well as the premotor cortex (BA 6), suggesting greater activity in these putative MNS regions when spontaneous mimicry was unrestricted compared to when it was not so. While the mechanism through which IFG and other MNS regions facilitate mimicry remains unresolved, suggested routes include the decomposition of an observed action into simpler parts and subsequent encoding of action sequences for detailed and accurate simulation (Molnar-Szakacs et al., 2006).

Specifically in regions involved in reward processing, we found a significant Tongue × Emotion interaction in bilateral OFC, driven by significantly greater activity in the NoTongue Happy vs. Tongue Happy condition, while there was no significant difference in activity between the NoTongue Angry vs. Tongue Angry conditions. The OFC is a key structure within the brain's reward systems (Gottfried et al., 2003, O'Doherty et al., 2001) and projects directly to the nucleus accumbens of the VS (Everitt and Robbins, 2005, Haber and Knutson, 2010). The region has been linked to empathic behaviour (Rankin et al., 2006) and to emotion processing (Beer et al., 2006, Blair and Cipolotti, 2000, Hornak et al., 1996). It has been suggested that activity in the OFC may reinforce the value of sensory stimuli based on its accompanying visceral sensation (Rankin et al., 2006). In the context of the current study, this result suggests that the unrestricted spontaneous mimicry of happy faces may be associated with positive valuation (i.e., ‘reward’) in the OFC. As a corollary, restricting spontaneous mimicry would block this positive valuation effect. Thus, the findings from the current study lend some support to the hypothesis that the spontaneous mimicry that occurs in social interaction is rewarding and that this reinforcement may motivate similar behaviours in the future. This result also supports the hypothesis from primate literature suggesting an intrinsic reward value for the act of mimicking (de Waal and Bonnie, 2009).

Contrary to our prediction, there was no Tongue × Emotion interaction in either the right or left VS. Specifically, whilst there was the expected increase in VS activity in the NoTongue Happy vs. Tongue Happy condition, there was also an unexpected increase in activity in the NoTongue Angry vs. Tongue Angry condition. While it is difficult to interpret a null result, one potential explanation is offered. First, it is possible that some of the striatal signal was lost when movement-related components were removed by ICA. In view of the role of the dorsal striatum in motor control, the low spatial resolution of fMRI for these deep structures, and that the ‘Tongue’ condition involved more such activity, it is possible that some of the VS signal was lost while eliminating the components that had high dorsal striatal (motor control-related) signal.

The analysis described above provides an insight into how spontaneous mimicry of happy faces activates one of the key nodes of reward processing. In conjunction with the findings in Sims et al. (2014), and Neufeld and Chakrabarti (2016), the results from the current study support the hypothesis that a bidirectional link exists between the brain's reward and mimicry systems. Future studies should test whether this link from mimicry to reward holds true for other emotions as well, such as angry faces. Such an experiment will require an experimental manipulation such that the movement of the corrugator supercilii is selectively restricted in one condition, without having any impact on the ZM.

Individual differences in the strength of this reward-mimicry link were tested further by examining the association between trait empathy and the bilateral OFC response to mimicry. Specifically, we found a significant positive correlation between the Tongue × Emotion interaction contrast values in the bilateral OFC and the IRI score. In line with our hypothesis, individuals with higher trait empathy were found to have stronger OFC activity associated with spontaneous facial mimicry. This is consistent with earlier observations of greater ventral striatal response in individuals with higher trait empathy, in response to social rewards such as happy faces (Chakrabarti et al., 2006, Gossen et al., 2014). Janowski et al. (2013) have shown that empathic choice is determined by processing of value in the ventromedial prefrontal cortex in a choice task. It is possible that lower scores on trait empathy might represent lower levels reward for the act of mimicry. This result echoes the behavioural findings that show a reduced reward value for being mimicked in individuals with low trait empathy (Neufeld and Chakrabarti, 2016).

We mention the results of the whole-brain exploratory analysis below for the sake of completeness. A positive correlation between the interaction contrast and the IRI score was noted in the right mid-cingulate cortex (MCC). This region has been previously reported by a meta-analysis as a key region involved in empathy for pain (Lamm et al., 2011), and has also been associated with empathy for personal distress (Yamada and Decety, 2009). The same analysis revealed significant clusters in the right temporoparietal junction, dorsomedial prefrontal cortex, and left anterior temporal lobe, all of which regions have been reported in previous meta-analysis of Theory of Mind processes (Schurz et al., 2014). While any speculation on the role of these regions relies on reverse inference by definition, it is possible that individuals high in trait empathy potentially engage in a greater degree of mentalizing when they are allowed to mimic faces spontaneously. Future studies could examine this possibility directly, by probing, e.g. if restricting spontaneous facial mimicry impairs performance in a Theory of Mind task.

Two caveats should be considered while interpreting the results of the study. First the sample size of the pilot study demonstrating the manipulation check (i.e. lower ZM response in the Tongue compared to the NoTongue condition) is small. Future studies could build on the current paradigm by collecting simultaneous facial EMG data from the ZM inside the MRI scanner. Second, the number of individuals included in the correlation analysis is lower than the total number of participants. This data loss was unavoidable due to participants who did not complete the questionnaire despite repeated reminders. Future studies should test this paradigm in larger samples, allowing for such participant dropout.

### 4.1 Conclusion

Using a validated task to restrict spontaneous mimicry of happy faces, we demonstrated the positive relationship between spontaneous facial mimicry and reward processing. Importantly more empathic individuals showed a greater reward-related neural response to mimicry, consistent with similar results from earlier behavioural studies. Future studies should extend these paradigms to conditions associated with low trait empathy, such as Autism Spectrum Disorders (ASD). These results raise the question of whether training of facial mimicry can enhance social reward processing in individuals with ASC.
